# Mechanical Properties of Human Dura Mater in Tension – An Analysis at an Age Range of 2 to 94 Years

**DOI:** 10.1038/s41598-019-52836-9

**Published:** 2019-11-13

**Authors:** Johann Zwirner, Mario Scholze, John Neil Waddell, Benjamin Ondruschka, Niels Hammer

**Affiliations:** 1 Department of Anatomy, University of Otago, Dunedin, New Zealand; 2 Institute of Materials Science and Engineering, Chemnitz University of Technology, Chemnitz, Germany; 3 Sir John Walsh Research Institute, Faculty of Dentistry, University of Otago, Dunedin, New Zealand; 4 Institute of Legal Medicine, University of Leipzig, Leipzig, Germany; 5 Department of Orthopedic and Trauma Surgery, University of Leipzig, Leipzig, Germany; 6 Fraunhofer IWU, Dresden, Germany; 7 Department of Anatomy, Medical University of Graz, Graz, Austria

**Keywords:** Neuroscience, Anatomy, Materials science

## Abstract

Realistic human head models are of great interest in traumatic brain injury research and in the forensic pathology courtroom and teaching. Due to a lack of biomechanical data, the human dura mater is underrepresented in head models. This study provides tensile data of 73 fresh human cranial dura mater samples retrieved from an area between the anterior middle and the posterior middle meningeal artery. Tissues were adapted to their native water content using the osmotic stress technique. Tensile tests were conducted under quasi-static uniaxial testing conditions with simultaneous digital image correlation. Human temporal dura mater is mechanically highly variable with regards to its elastic modulus of 70 ± 44 MPa, tensile strength of 7 ± 4 MPa, and maximum strain of 11 ± 3 percent. Mechanical properties of the dura mater did not vary significantly between side nor sex and decreased with the age of the cadaver. Both elastic modulus and tensile strength appear to have constant mechanical parameters within the first 139 hours post mortem. The mechanical properties provided by this study can help to improve computational and physical human head models. These properties under quasi-static conditions do not require adjustments for side nor sex, whereas adjustments of tensile properties accompanied with normal aging may be of interest.

## Introduction

**Figure 1:**
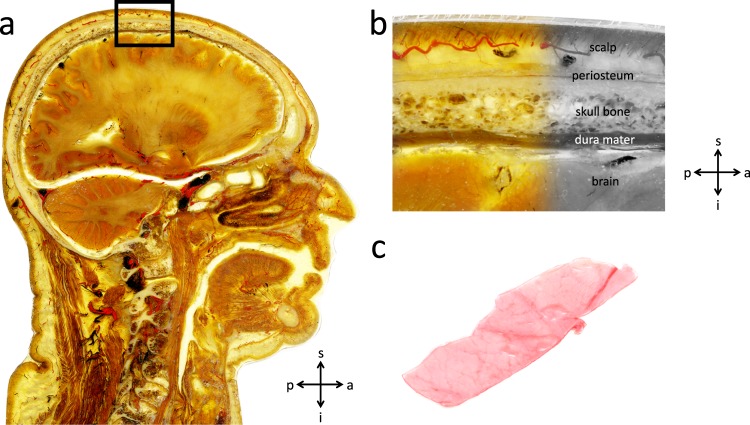
(**a**) An epoxy resin (E12) plastinate showing a sagittal section of the human head displaying the complex anatomy that has to be simulated in human skull models (scale bar 10 mm). (**b**) Higher magnification of the black square placed in *a*. The different layers of the skull can be identified. (**c**) Brain facing surface of human dura mater sample. s, superior; a, anterior; i, inferior; p, posterior.

Traumatic brain injury (TBI) is a major burden for public health care systems world-wide with about 2.8 million emergency department visits and 56,000 deaths in the United States or 82,000 througout Europe annually^1,2^. Injury to the head is the most common cause of death in physical attacks, intentional self-harm and accidents predominantly of younger adults^1,3^. The underlying mechanisms of the head injury (e.g. blunt vs. sharp trauma, non-penetrating vs. penetrating trauma, acceleration-deceleration vs. rotational trauma or impact damage vs. diffuse axonal damage) are required to be settled without a doubt in courtrooms to discharge or convict suspects of a crime. In addition to these forensic questions, a thorough understanding of the mechanisms involved with head injury is essential for the determination of treatment in clinical care. Both computational and physical skull models can be used to reproduce forensic-relevant injuries in a reliable and bloodless manner, without the ethical concerns that would be present in cadaver or animal experiments^4–9^. Carr *et al*. showed that by including the refined details of the anatomical head structure, such as modelling the brain in an anatomical correct manner, the skull fracture patterns tended to be more realistic^10^. Besides the anatomical accuracy, a lifelike imitation of biomechanical tissue properties is crucial for realistic skull models (The complex human head anatomy comprised of a number of layers and the position of the dura mater are displayed in Fig. 1a,b). Polyurethane resins and ten percent gelatin have been shown to be realistic replacement materials, as they resemble the biomechanical properties of the cranium and brain, respectively^4,11^. Furthermore, Thali *et al*. reproduced tissue bridges and a periosteum pocket, after applying blunt forces to their skin-skull-brain model, using silicon as a skin replacement material and latex for the periosteum^4,12^. Despite their important role in shear stress reduction, intracranial pressure reduction and the mitigation of the dynamic brain response under blast loading in TBI, the meninges are mostly neglected in physical and computational skull models to date^6,7,12–15^. The frequent negligence of the dura mater in human skull models may be a result of the lack of biomechanical basis data of human dura mater which includes only a very limited number of investigated specimens^16–21^. Human head models which include the dura mater involve a wide range of biomechanical properties as reflected by previous studies reporting an elastic modulus between 16 and 130 MPa^8,22^. The purpose of this study is to provide basic biomechanical data of human temporal dura mater obtained from 73 cadavers in a fresh and chemically unfixed condition.

In this current biomechanical study utilizing human dura mater we addressed the following hypotheses:

1. Elastic modulus, ultimate tensile stress and maximum strain of human dura mater change with age. The relationship between age and biomechanical properties of the dura mater has to date only been investigated by one group before with a sample size of 20 cadavers^18^.
2. Elastic modulus, ultimate tensile stress and maximum strain of dura mater do not differ significantly between sexes. Morphological sex-related differences of the structures surrounding the dura mater might influence the dura mater biomechanically. These have only been shown on the dura unrelated outer surface of the temporal bone^23^.
3. The elastic modulus of human dura mater increases and ultimate tensile load decreases with longer post-mortem delay (PMD), due to cellular autolysis and decomposition after death.

## Materials and Methods

### Acquisition and processing of human dura mater samples

126 human dura mater samples according to Fig. 1c were removed from temporal bone mostly bilaterally, after opening the skulls from 75 cadavers (50 ♂, 25 ♀) with a mean age of 50 years (range 2–94 years) during forensic autopsies at the Institute of Legal Medicine, University of Leipzig, Germany. Tissues were obtained in a fresh condition (without any chemical fixation). The samples were harvested from an avascular area located between the anterior middle meningeal artery and the posterior middle meningeal artery. Prior to the autopsy, the cadaves were stored at 4 °C upon arrival at the mortuary. The PMD at the time of tissue retrieval averaged an estimate of 75 ± 30 hours (range 11–146 hours), with variations as a result of the timing of the autopsy. The mean thickness of the specimens was 0.68 ± 0.20 mm (range 0.40–1.40 mm). All samples were kept frozen at −80 °C for storage following an initial precooling stage. The study was approved by the ethics committee of the University of Leipzig, Germany (protocol number 486/16-ek). Institutional approval for the use of the post-mortem tissues was obtained from the Institute of Anatomy, University of Leipzig, in line with the Saxonian Death and Funeral Act of 1994 (third section, paragraph 18 item 8) as part of an ongoing donation program.

### Osmotic stress protocol for water content adjustment

**Figure 2:**
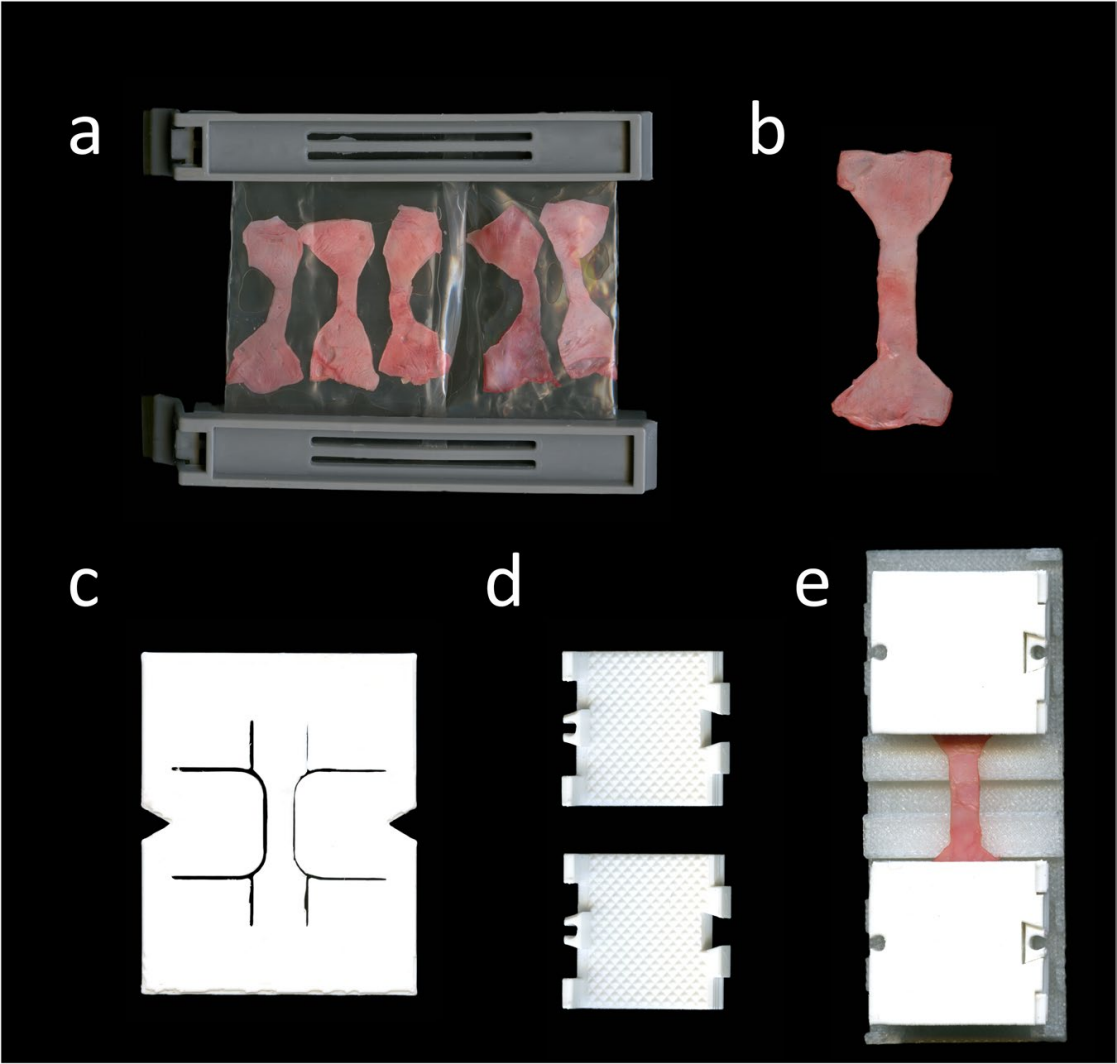
Images of equipment for experiemental setup (**a**) Tapered specimens clipped in a dialysis membrane of 64 mm (Spectra/ Por®; molecular weight cut off 6–8 kDa) for osmotic adaptation. The samples were submerged in a 5.0 wt-percent/0.02 wt-percent Tris-PEG solution on a shaking table at 4 °C for 24 hours prior to the tensile tests. (**b**) Specimen after layout following the tapering with the template shown in c. (**c**) To ensure comparable testing conditions specimens were tapered uniformly using a 3D-printed template adapted from the ISO 527-2 standard. (**d**) 3D-printed clamps with pyramids at their contact surface to the specimen were used to minimize material slippage when testing. (**e**) The specimen is mounted with clamps. A 3D-printed desk is used to assure consistent clamp-to-clamp distances in all specimens.

The osmotic stress technique was applied according to a technique described previously^24^. Initially, sections of dura mater from 30 cadavers were removed and sectioned into smaller pieces (10 subsamples per cadaver from 30 cadavers, 300 samples in total). One sample of each cadaver was used to determine the initial (fresh) water content, totalling 30 samples. The remaining 270 samples were assigned randomly over the 1-, 2-, 4-, 8- and 24-hour time frames at 20 mM hydroxymethyl aminomethane-buffered polyethylene glycol (Tris-PEG; pH = 7.4; Merck KGaA, Darmstadt, Germany; molecular weight 20,000 kDa) solutions of 2, 3, 4 and 5 percent, respectively (Fig. 2a). In order to determine the water content, the difference between wet weight and dry weight (following lyophilization) was determined. For the PEG-adjusted samples, the wet weight was defined as the weight following the adjustment of the water content in the given concentration for the given time following the removal from the dialysis membrane. The lyophilisation was done at 20 °C.

### Mechanical testing, data processing and scanning electron microscopy

**Figure 3:**
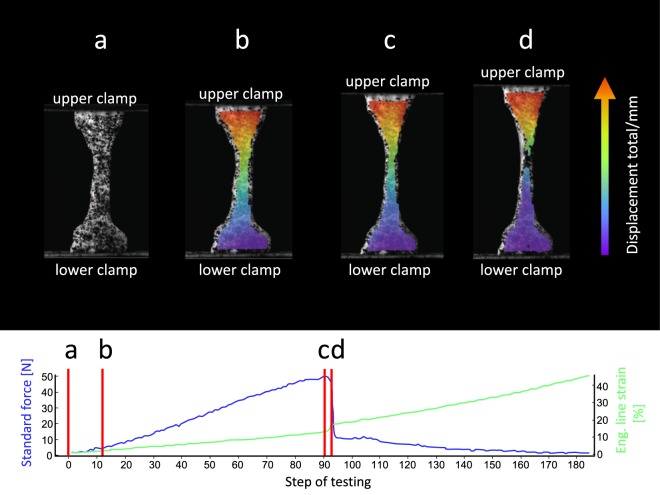
Images of clamped human dura mater specimen generated with digital image correlation software at different steps of the uniaxial tensile testing. The related force-curve (blue curve) and nominal strain-curve (green curve) are displayed. The straight red lines reflect the testing moment shown in the related image displayed above. During the uniaxial test, the upper clamp moves away from the fixed lower clamp and thus strains the clamped specimen. (**a**) The speckled specimen is clamped in the testing machine. (**b**) Specimen at the beginning of uniaxial tensile loading. (**c**) With load increase the tapered area deforms uniformly to the maximum of the stress-strain curve. (**d**) A localized failure in the tapered area leads to a rapid drop of the stress-strain curve.

Avascular dog bone-shaped dura mater samples as shown in Fig. 2b were removed from the harvested material, adapted to the ISO 527-2 standard (International Standard Organization, 1996, Fig. 2c). The area of parallel measurement length of the tapered specimens was casted with polysiloxane impression material (medium-bodied, Exahiflex, GC Corporation, Tokyo, Japan) for the analyses of the cross sections. The cross-section casts were then scanned at 1200 dpi resolution (Perfection 7V750Pro, Seiko Epson Corporation, Suwa, Japan). Measure 2.1d software (DatInf GmbH, Tübingen, Germany) was used to compute the cross sectional areas. To create a random grey intensity distribution essential for digital image correlation (DIC) specimens were speckled before tensile testing (Fig. 3a). Tensile tests were performed in a universal testing machine (Zwick Roell 20 kN Allround Table Top Z020, Ulm, Germany) at room temperature. 3D-printed clamps as shown in Fig. 2d,e were used to minimize specimen slippage during the testing as has been evaluated recently^25^. Preconditioning of the tissue was performed by applying 20 load-unload cycles at a force range of 0.5 to 2.0 N. Subsequently, the tissues were stretched until failure (Fig. 3b–d). An Xforce P load cell of 2.5 kN (accuracy grade 1 defined by ISO 7500) was used with testControl II measurement electronics (all Zwick Roell). The crosshead displacement rate of 20 mm/min and a sampling rate of 100 Hz were used for the force readings. Surface deformation was recorded perpendicular to the surface by a DIC system using a single charge-coupled camera with a 2.8 Megapixels resolution (Q400, Limess, Krefeld, Germany). Strain data during mechanical testing was evaluated with the ISTRA 4D software (VRS 4.4.1.354, Dantec Dynamics, Ulm, Germany) and an engineering strain was calculated between two points in the parallel zone of each sample during testing. Engineering stress-curves were furthermore calculated from the synchronized force readings and the measured cross-sectionional areas using MATLAB R2017b software (Mathworks, Natick, MA, USA). Under inclusion of the synchronized nominal strain from DIC the stress-strain-curves were plotted. The ultimate tensile strength was defined as the maximum force divided by the initial cross-sectionional area. The elastic modulus was evaluated in the linear part of each nominal stress-strain curve by a regression analysis. Maximum force (F_max_) was defined as the highest value of each force curve and maximum strain was defined as the strain of the sample when reaching the F_max_.

Statistical data evaluation was done with Excel Version 16.15 (Microsoft Corporation, Redmond, WA, USA) and GraphPad Prism software version 7 (GraphPad Software, La Jolla, CA, USA). P-values equal to or smaller than 0.05 were considered to be statistically significant. The plus-minus sign (±) reflects the standard deviation for values with a Gaussian distribution and the standard error of the mean for values that were not distributed normally (distribution tested by D’Agostino-Pearson omnibus normality test). Additionally, scanning electron microscopy was performed of the arachnoid and bone surface layer of 10 temporal dura mater samples to investigate isotropy of collagens by means of a JEOL 6700 F field emission scanning electron microscope (JEOL, Peabody, MA, USA). The samples were coated in a K575X sputter coater with a 5 nm layer of gold palladium (Emitech Technologies, Kent, England).

**Figure 4:**
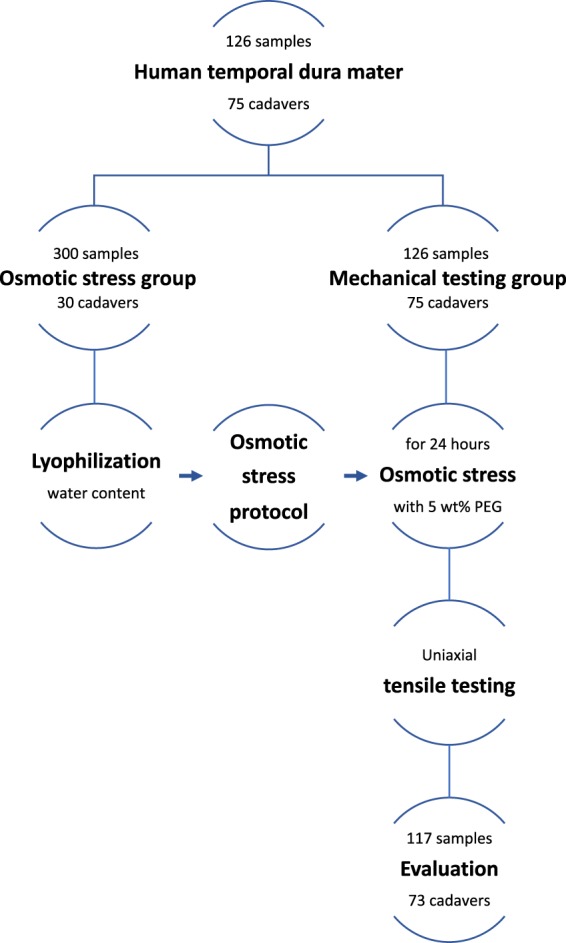
Flow chart summarizing the experimental setup. 117 samples survived the biomechanical testing and were evaluated. Due to the mostly bilateral testing of human temporal dura mater samples per individual cadaver, only two cadavers could not be included into the data evaluation.

The experimental setup used in this given study is visualized in Fig. 4.

## Results

### 5.0 wt-percent PEG was best suited to adjust the water content of human dura mater close to the fresh and unfixed condition

The native water content of human dura mater averaged 73 ± 8 percent by weight. The water contents after 24 hours in the 2.0, 3.0, 4.0 and 5.0 wt-percent PEG/0.02 wt-percent Tris solutions averaged 80 ± 9, 78 ± 3, 80 ± 7 and 75 ± 6%, respectively. A 5.0 wt-percent PEG/0.02 wt-percent Tris solution was chosen for tensile testing as it was closest to the native condition.

### The tensile properties of the human temporal dura mater are  highly variable between individuals

117 of the total 126 human dura mater samples (48 ♂, 25 ♀; 58 left temporal side, 59 right temporal side), with a mean age of 50 ± 23 years (range 2–94 years) were included in the data evaluation following the tensile testing. The nine specimens that were excluded from the data evaluation did not sustain the 20 preconditioning load-unload-cycles, resulting in premature failure (Fig. 3 displays an example of the stress-strain curves and DIC). The D’Agostino-Pearson omnibus normality test revealed Gaussian distribution for the PMD (p = 0.34), maximum force (p = 0.09) and the strain at maximum force (p = 0.81). Age (p ≤ 0.01), thickness of the dura mater sample (p ≤ 0.01), width of the tapered area of the dura mater sample (p = 0.047), elastic modulus (p ≤ 0.01) and tensile strength (p ≤ 0.01) were not distributed normally according to the D’Agostino-Pearson omnibus normality test.

**Figure 5:**
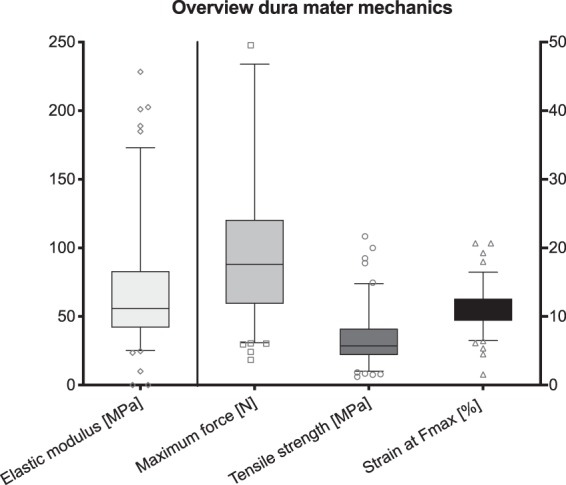
Overview of the tensile properties of human temporal dura mater (n = 117). The outlines of the boxes indicate the 25^th^ and 75^th^ percentile, the solid black line the median. Whiskers are defined as Tukey’s end of 1.5 times interquartile range and all outliers are illustrated as single marks outside these fences.

The obtained values from the mechanical tests were as follows: elastic modulus 70 ± 44 MPa, tensile strength 7 ± 4 MPa, maximum strain 11 ± 3 percent and maximum force 21 ± 18 N. The mechanical values were accompanied by high standard deviations (Fig. 5).

### Mechanical properties of the dura did not differ between side nor sex

**Figure 6:**
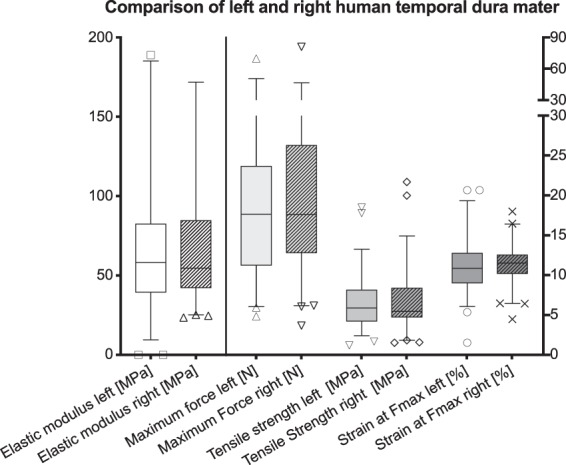
Mechanical properties of human dura mater samples are displayed separated according to the body side (left, n = 58; right, n = 59). The box-plots indicate no side-dependent differences in biomechanical answer at maximum force for human temporal dura mater in uniaxial tensile testing. The outlines of the boxes indicate the 25^th^ and 75^th^ percentile, the solid black line the median. Whiskers are defined as Tukey’s end of 1.5 times interquartile range and all outliers are illustrated as single marks outside these fences.

No side-dependent differences were observed for elastic modulus, tensile strength, maximum strain, nor F_max_ on a significant level. Also, the mean ranks of these four properties did not vary significantly between sides. Figure 6 summarizes the stress-strain and failure data.

**Figure 7:**
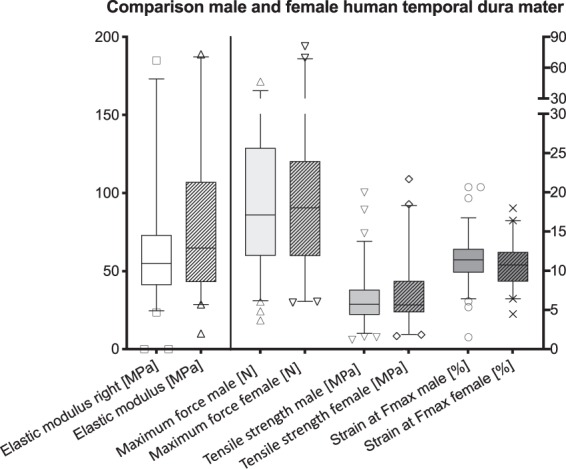
Comparison of male and female temporal dura mater. No statistically significant correlations were present between sexes. The outlines of the boxes indicate the 25^th^ and 75^th^ percentile, the solid black line the median. Whiskers are defined as Tukey’s end of 1.5 times interquartile range and all outliers are illustrated as single marks outside these fences.

The Kruskal-Wallis test yielded no differences in elastic modulus, tensile strength, maximum strain nor F_max_ between sexes on a statistically significant level (p > 0.05). Moreover, there was no statistical significant difference concerning male and female maximum forces of human temporal dura mater (Fig. 7).

### Mechanical properties of human dura mater decrease with age

**Figure 8:**
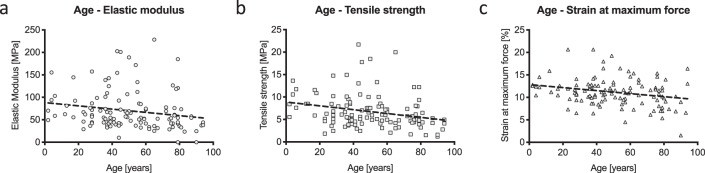
Mechanical properties of human temporal dura mater in relation to age. Weak negative correlations are depicted between elastic modulus (**a**), tensile strength (**b**), strain at maximum force (**c**) and age.

The Spearman correlation indicated statistically significant weak negative correlations between elastic modulus (r = −0.283; p = 0.002) and tensile strength (r = −0.299; p = 0.001) to the age of the deceased. Pearson correlation coefficient revealed a comparable weak negative correlation for the maximum strain (r = −0.229; p = 0.013) with the cadaver’s age at death. Figure 8 displays correlations between age and tensile properties graphically.

### Elastic modulus and tensile strength appear not to be altered within the first 139 hours post mortem as a consequence of storage in a non-frozen condition

The mean PMD of the evaluated dura mater samples was 74 ± 30 hours (range 11–139 hours). The mean PMD of the nine specimens that failed the test was not significantly higher with 93 ± 28 hours (range 52–146 hours; p = 0.064). One of the failed samples had a PMD larger than the here-defined maximum PMD of 139 hours. All samples with a PMD below this threshold were suitable for retrieving load-deformation data. No statistically significant differences were found concerning elastic modulus (r = −0.079; p = 0.395) and tensile strength (r = −0.031; p = 0.743) related to the PMD. Only the maximum strain was shown to be correlated to the PMD in a less intensity (r = 0.204; p = 0.027).

**Figure 9:**
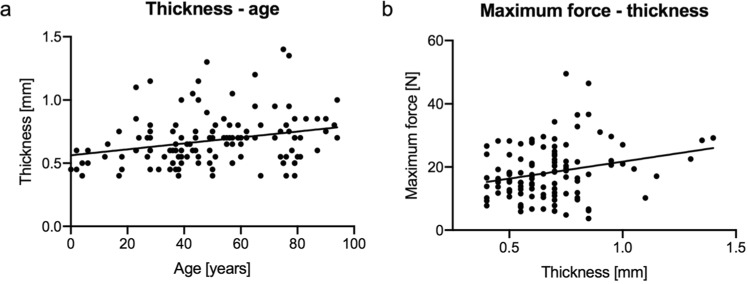
Age and maximum force in relation to thickness of the tested dura samples. Weak positive correlations were depicted for age (**a**) and maximum force (**b**) in relation to the thickness of the dura mater samples.

The thickness of the dura mater samples correlated with age and applicable maximum force. A weak positive correlation was observed between the thickness and the age of the cadaver at death (r = 0.288; p = 0.001). Of all tested mechanical parameters only the maximum force showed a significant correlation with the thickness (r = 0.221; p = 0.019). Figure 9 displays the correlations between thickness with age and maximum force.

### Scanning electron microscopy reveals differences in isotropy between different layers of the human dura mater

**Figure 10:**
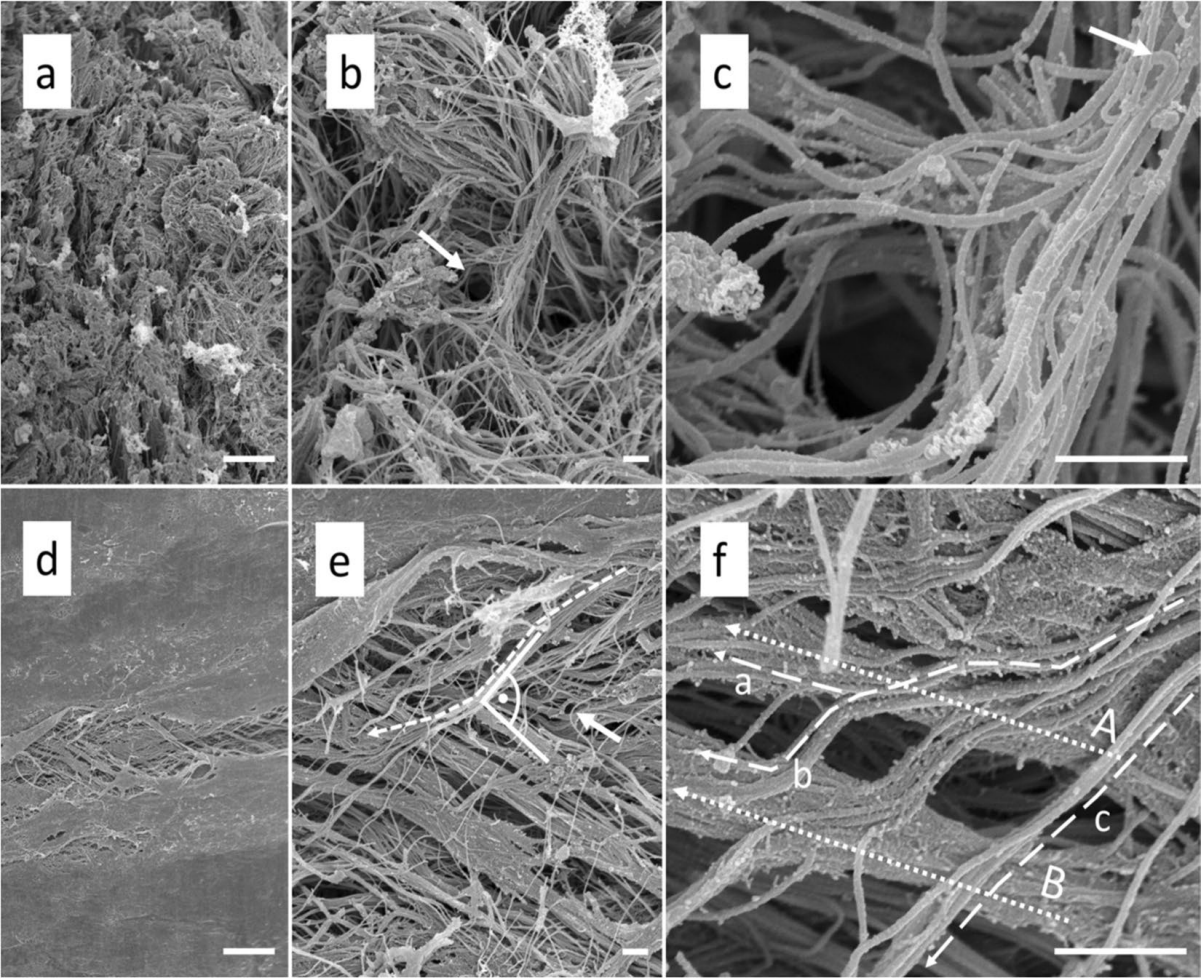
Scanning electron microscopy of human temporal dura mater samples. (**a**) The arachnoid layer of the temporal dura mater reveals an isotropic organization at 1000x magnification (scale bar 10 μm). (**b**) 5000x magnification of *a* shows that the collagens run in multiple directions in the arachnoid layer (scale bar 1 μm). The arrow points to a channel that might form the bed for a vessel in the native state. (**c**) The channel depicted in *b* is shown at a higher magnification of 25,000 × (scale bar 1 μm). Note the bending of the collagen fibres (arrow) in proximity to the vessel channel. (**d**) The bone surface layer of the human temporal dura reveals a clear organization of the collagens at 1000x magnification (scale bar 10 μm). (**e**) A 5000x magnification of *d* displays the anisotropic structure of the bone surface layer (scale bar 1 μm). Collagen bundles are running in a preferred direction. The bone surface layer of the temporal dura is composed of several superimposed collagen layers which run into different directions with angles up to 90°. The arrow marks a single fiber, which is most likely elastin due to its single course crossing several subjacent collagen bundles (for the characteristics of elastic fibres in the SEM see Woplers^47^). (**f**) A 25,000x magnification of *e* shows the splitting of the collagen bundle marked with a dotted line in *e* (scale bar 1 μm). Note how the fibres of the splitting bundle either join collagen bundles A (dotted line a) and B (dotted line b) or just pass the bundles on their way (dotted line c).

The arachnoid layer of the human temporal dura mater mostly showed a random collagen distribution (Fig. 10a). Therefore, the arachnoid layer may be considered to be isotropic from a morphological distribution perspective. In the arachnoid layer, fiber alignment was seen at sites where vessels pass the layer (Fig. 10b,c). The bone surface layer revealed a high collagen alignment with several superimposed layers, which run almost perpendicular one to the other (Fig. 10d,e). The bone surface layer may therefore be considered anisotropic regarding its collagen arrangement.

## Discussion

Human dura mater is a five-layered membranous tissue consisting of a compact and dense collagen mesh with a few scattered fibroblasts and elastin fibres embedded in a mucopolysaccharide-water matrix^17,20,26^. In general the human cranial dura mater is considered to be an isotropic material^17,27^. However, areas of structural anisotropy of the human cranial dura mater have been shown before^27^. The scanning electron microscopy performed in this study confirmed a relatively uniform alignment of collagen fibres in the bone surface layer of the temporal dura mater samples, forming an argument in favor of the anisotropic behavior of these layers. To keep the unswayable factors as comparable as possible, dura mater samples of an avascular region between the anterior middle meningeal artery and the posterior middle meningeal artery were used in this study as in general dura mater of the temporal area has a reduced vasculature, a uniform thickness and is less firmly attached to the skull bone^17^. Furthermore, pathophysiological straining of dura mater seems to be highest at temporal sites in TBI cases given that epidural hemorrhages are mainly associated to ruptures of the middle meningeal artery^28^. Realistic skull models can be used in the courtroom to verify or disprove forensic hypotheses. For instance a ballistic or blunt force experiment using such skull models could be conducted according to the presented case or the testimony of the accused and subsequently compared to radiographic or autopsy images of the related case. Furthermore, forensic pathology teaching and research profit from more realistic human skull models by means of demonstrative experiments in classrooms and laboratories^9,29,30^. Detailed mechanical data of the tissues of the human head is to date missing in large numbers and in a chemically fresh condition. Apart from legal medicine, especially in traffic and road accidents, both computational and physical skull models have evolved becoming a promising tool to investigate TBI mechanisms, its pathogenesis and potential measures for prevention^13^. It has been estimated that TBI leads to mortality or hospitalization of about 10 million people in the world annually^31^. Therefore, it is considered to be a critical public health and socio-economic problem throughout the entire world^32^. Efforts how to prevent TBI by improvements in head protection devices such as intelligent helmets are promising as it has been shown that treatment options are still vastly limited to reduce so-called secondary brain damages in spite of the advances in neurology and neurosurgery^33^. Biomechanically validated and morphologically realistic models of the human head are an emerging experimental tool to achieve advances in head protection and lower the related number of severe TBIs in the future^34^. Gu *et al*. investigated the effects of the meninges on the dynamic response to brain tissue in a finite element analysis by referring head models with and without the meninges to blasting loads. They found that the meninges migitate the dynamic response of the brain tissue, which underlines the need to respect the dura mater in human head models. Moreover, by applying Ogden and elastic models Gu *et al*. showed that the intracranial pressure and strain response are related to the properties of the material used for dura replacements. This highlights the demand for an accurate biomechanical dura mater description, including its relation to factors such as age, sex and PMD, which is provided in this study^15^.

Constitutive biomechanical data of the human cranial dura mater is scarce and has been investigated by only a few groups to date^16–21^. Galford and McElhaney did not specify the number of their investigated dura mater samples^16^. Twenty-two human dura mater samples is to date the highest investigated number, which obtains basic biomechanical data such as ultimate tensile strength, elastic modulus, incremental modulus or strain at fracture^21^. In our study the elastic modulus as an approximation of a viscoelastic and time-dependent material behavior, ultimate tensile strength and maximum strain of human temporal dura mater did not vary significantly between sides. Recently, consistent with our results De Kegel *et al*. noted that a significant site dependency of biomechanical cranial dura mater data could not be detected^19^. Due to the side symmetry of the brain and the surrounding skull a side difference in biomechanical dura mater properties may not be expected. Elastic modulus, ultimate tensile stress and maximum strain of human temporal dura decrease with ageing. An age-dependent decrease in tensile strength of human dura mater tissue was stated before^18^. In this study we observed a moderately increasing thickness of temporal dura mater with age. An age-dependent thickening has been stated for spinal dura mater before and was related to fibrous degeneration^35^. Recently, a study by Fam *et al*. observed a negative correlation of age with dural thickness of the skull base^36^. Therefore, it might be that the thickness of human cranial dura mater does not generally increase or decrease, but change site-dependently with age. Elastic modulus, ultimate tensile strength and maximum strain of human temporal dura mater did not differ significantly between sexes. Sex dependency of constitutive biomechanical human dura mater properties have not been reported before to the best of our knowledge.

The human temporal dura mater is an inter-individually highly variable structure concerning variations of the mean values of elastic modulus, ultimate tensile strength  and maximum strain of 61, 56 and 28 percent, respectively (Fig. 5). The high variations in the mean values may partly be explained by anisotropic behavior of the dural sub-layers, as was shown for the bone surface layer in this study. Hamann *et al*. stated from own unpublished results that fibers which have a parallel alignment were stronger than perpendicular orientated ones in uniaxial tensile testing^27^. We conclude from the high variation of mean values and the obtained anisotropic behavior of the bone surface layer that the human temporal dura mater is an anisotropic material with implications on the biomechanical properties obtained in uniaxial tensile tests. The elastic modulus obtained in our study averaged 70 MPa. Galford and McElhaney obtained a lower Young’s modulus of 32 MPa for the human dura mater in a dynamic testing setup^16^. This value is widely used to represent the dura mater in computational finite element models of the human head to investigate various impact scenarios^37–43^. Van Noort *et al*. reported a low incremental modulus of 29 MPa^17^. In contrast the McGarvey *et al*. group stated a tissue modulus of 62 MPa^20^, which appears to be the most similar result compared to our findings. A more recent investigation of 22 human dura mater samples of the posterior cranial fossa harvested from predominantly elderly fresh cadavers, showed elastic moduli between 19 and 91 MPa. This is in line with our results of temporal dura mater^21^. As mentioned for elastic modulus, the tensile strength of 7 MPa reported in our study shows a high variation and lower values than published previously. McGarvey *et al*. showed in their experiments a higher ultimate tensile strength of 9 MPa^20^. We report a strain at failure of 15 percent. Similar results were stated by van Noort *et al*. with a failure strain of 18 percent, whereas the strain at fracture of 32 percent reported by McGarvey was almost twice as high^17,20^. Our data is in line with those of van Noort *et al*., who mentioned a considerable variation in the mechanical properties of human dura mater tissue^17^. We consider the composition of the dura mater as the main factor influencing the high inter-individual variation of the tensile properties. Collagen fibers were shown to be organized in multiple directions within the human dura mater leading to a structural isotropy of the tissue, which could be partly confirmed for the arachnoid layer in this study. However, as shown on the bone surface layer in the scanning electron microscopy in the given study, and also shown by Hamann *et al*., using small angle light microscopy, 80 percent of samples investigated had highly aligned collagen fibers in the temporal region of human dura mater^27^. As the human dura mater has a macroscopically isotropic appearance when harvested priority was given to the obtainment of avascular layouts of human dura mater samples for tensile testing. Therefore, it must be considered that areas of anisotropy tend to differ in tensile properties among the tested samples, due to a more defined collagen fiber architecture. The PMD of the investigated cadavers and the resulting specimens did not affect the tensile properties significantly, in an approximated time frame of 6 days post mortem. To our knowledge, this study forms the first attempt to investigate PMD-related alterations in dura mater mechanics. Further to that, *in-vivo* models in comparable species are lacking. Studies that investigated human lumbar dura mater or dura mater of other species are beyond the scope of this manuscript as our work focuses on human cranial dura mater to provide basic data for human skull model studies^44–46^. Therefore, from a biomechanical perspective we conclude that fresh human dura mater can be harvested and used as a transplant for a minimum of at least 5 days after death, if the cadaver was stored under normal cooling conditions at +4 °C. Human dura mater might be less prone to degradation compared to other tissues as it is a nearly acellular material^18^.

A number of limitations have to be addressed in this given study. Mechanical testing of the human dura mater samples was performed under uniaxial loading and quasi-static conditions. This should be considered when the data is used in context of impacts to the human head, which are often of a dynamic and multiaxial loading nature. As the dura mater is attached to the skull it cannot be excluded that cells and extracellular matrix layers of dura mater were still attached to the bone by means of Sharpey fibers during the harvesting under dissection conditions with the naked eye. Further, in spite of the given sample size, larger specimen numbers or other dura mater localizations would help account for anisotropic and disease dependent alterations in more detail. We could not show any obvious signs of deterioration of the dura samples in the given PMD interval, but alterations of the tissues post-mortem may have still mimicked minute differences in the dura mater mechanics in spite of our standardization efforts. It has not been histologically investigated, if the 24 hours of cooled PEG treatment led to an increased tissue deterioration.

## Conclusions

Fresh human cranial dura mater is an inter-individually variable material concerning its tensile properties (elastic modulus, tensile strength and maximum strain) under uniaxial quasi-static loading. These three parameters decrease with age and do not differ according to sex and side. Within 139 hours after death, human cranial dura mater did not show any signs of tissue deterioration, when stored under ambient conditions at 4 degrees celsius.

## Data Availability

The datasets generated and analysed during the current study are available from the corresponding author on reasonable request.
